# Recurrent Myocarditis in Young Adults: Identifying High-Risk Phenotypes and the Protective Effect of Adequate Colchicine Therapy

**DOI:** 10.3390/medicina62071323

**Published:** 2026-07-09

**Authors:** Bihter Senturk, Tugce Colluoglu, Cisem Oktay, Adam U. F. Turk, Ilerya Balikoglu, Mehmet Yavuz, Mehmet Kis, Huseyin Dursun, Mehmet Birhan Yilmaz

**Affiliations:** 1 Department of Cardiology, Faculty of Medicine, Dokuz Eylül University, 35340 Izmir, Türkiye; tugcecolluoglu48@gmail.com (T.C.); cisem.oktay@deu.edu.tr (C.O.); ilerya.b@gmail.com (I.B.); mehmet.kis@deu.edu.tr (M.K.); drhuseyindursun@gmail.com (H.D.); birhan.yilmaz@deu.edu.tr (M.B.Y.); 2 Central Hospital Izmir, 35540 Izmir, Türkiye; adamusamaturk@hotmail.com; 3 Department of Cardiology, Faculty of Medicine, Mersin University, 33343 Mersin, Türkiye; mehmetyavuz@mersin.edu.tr

**Keywords:** recurrent myocarditis, young adults, electrocardiographic markers, late gadolinium enhancement, colchicine therapy

## Abstract

*Background and Objectives*: Recurrent myocarditis confers a significant prognostic burden yet lacks validated predictive markers to guide risk stratification. This study aimed to identify independent clinical, electrocardiographic, and cardiac magnetic resonance (CMR) determinants of recurrence at a median of 7 days (IQR: 3–14) after the index event in young adults with acute myocarditis and to evaluate the role of colchicine therapy duration in modulating recurrence risk. *Materials and Methods*: This retrospective observational cohort study enrolled 162 patients admitted with a diagnosis of acute myocarditis to a tertiary cardiology center between January 2014 and January 2024. Diagnosis was established according to ICD-10 criteria with confirmation by CMR. The primary endpoint was recurrent myocarditis at two-year follow-up. Independent predictors were delineated through binary logistic regression and time-to-event analyses using multivariate Cox proportional hazards modeling. *Results*: The two-year recurrence rate was 12.3%, with a median time to recurrence of 13.5 months. Time of colchicine use, ST-segment depression on the admission electrocardiogram (OR 14.469, 95%CI 2.416–86.673; *p* = 0.003), and late gadolinium enhancement (LGE) on CMR (OR 10.362, 95%CI 1.614–66.549; *p* = 0.014) emerged as independent predictors of recurrence. Colchicine therapy sustained for a minimum of three months was independently associated with a markedly reduced recurrence risk (OR = 0.295, 95%CI = 0.098–0.891; *p* = 0.030). Cox regression corroborated these associations, demonstrating substantially elevated hazard ratios for ST depression (HR = 10.729, 95%CI = 2.201–52.298; *p* = 0.003) and LGE (HR = 8.064, 95%CI = 2.036–31.942; *p* = 0.003), with a protective effect of adequate colchicine duration (HR = 8.577 for treatment cessation; *p* = 0.025). A multisegmental LGE pattern and index episode onset during the winter months were additionally associated with recurrence. *Conclusions*: ST-segment depression on admission electrocardiography and CMR-detected LGE may represent potent, independent predictors of myocarditis recurrence. Adequate colchicine duration of at least three months may attenuate recurrence risk, underscoring the critical importance of optimizing anti-inflammatory therapy duration and integrating electrocardiographic and imaging phenotyping into personalized long-term surveillance strategies.

## 1. Introduction

Myocarditis is an inflammatory disease affecting the myocardium, diagnosed based on established histological, immunological, and immunohistochemical criteria [1]. Its etiology is broadly divided into infectious causes, most commonly viral; non-infectious causes, including systemic autoimmune disorders, drugs, and toxins; and an underlying genetic predisposition. Myocardial injury is thought to evolve from an initial phase of direct myocyte damage and innate immune activation toward an adaptive, frequently autoimmune phase, with eventual recovery or progression to fibrosis [2,3]. The clinical presentation is heterogeneous, ranging from chest pain to new-onset heart failure, arrhythmias, syncope, and cardiogenic shock [1,4]. The 2024 ACC Expert Consensus Decision Pathway defines acute myocarditis as occurring within one month of symptom onset and proposes a four-stage classification integrating clinical, cardiac magnetic resonance imaging (CMR), and biopsy findings [4]. Potential complications include heart failure, ventricular arrhythmias, sudden cardiac death, and disease recurrence [4].

Recurrent myocarditis, while relatively infrequent in clinical practice, carries profound prognostic implications and complicates long-term therapeutic management [5,6]. Data from observational cohorts have identified several predictors of recurrence, including prior myocarditis episodes, younger age at initial presentation, and a complicated clinical trajectory during the index hospitalization—often marked by prolonged inpatient stays or the occurrence of ventricular arrhythmias. Additionally, concurrent systemic inflammatory or autoimmune comorbidities and a documented viral prodrome at the time of diagnosis remain reproducible determinants of disease relapse [5,6].

Despite progress in non-invasive diagnostic methods and risk stratification tools, neither initial cardiac imaging parameters nor traditional clinical phenotyping at presentation seems to effectively predict the risk of recurrence. This highlights the importance of focusing on detailed patient history and the immunological environment as essential yet underused approaches for identifying individuals at higher risk of disease relapse [7]. Consequently, the present study sought to identify clinical and imaging determinants of susceptibility to recurrence, with the ultimate goal of tailoring personalized, long-term surveillance and management strategies.

## 2. Materials and Methods

### 2.1. Data Source and Study Design

This research was a retrospective observational cohort study of patients with a history of myocarditis, admitted to the Department of Cardiology at Dokuz Eylul University, Faculty of Medicine, from 1 January 2014 to 1 January 2024. We conducted a search and extracted data from the hospital information management system of Dokuz Eylul University Faculty of Medicine Hospital (protocol code 2024/41-02 and date of approval 11 December 2024).

The diagnosis of myocarditis was based on ICD-10 codes I40.0 (infective myocarditis), I40.1 (isolated myocarditis), I40.8 (other acute myocarditis), and I40.9 (acute myocarditis, unspecified), along with the presence of symptoms, elevated cardiac biomarker levels (high-sensitivity cardiac troponin [hs-cTn] and/or creatine kinase-MB [CK-MB]), index echocardiographic and coronary angiographic findings, and subsequent validation by the CMR. The median time from the diagnosis of acute myocarditis to CMR was 7 days, with an interquartile range of 3 to 14 days. Recurrent myocarditis was documented by the hospital discharge reports of the patients with a history of myocarditis from the electronic healthcare system. The diagnosis of recurrent myocarditis was confirmed using the same criteria as the first myocarditis episode. We excluded patients with acute coronary syndrome, including ST-elevation myocardial infarction, non-ST-elevation myocardial infarction, and unstable angina pectoris, confirmed by coronary angiography. Patients with a previous or current diagnosis of chronic coronary syndrome were excluded. All patients included in the current study had no evidence of overt coronary artery disease. Those over 50 years of age without accessible coronary angiography were excluded. Furthermore, patients above 70 years of age were excluded due to the inability of CMR to entirely exclude possible confounders [8].

Demographic variables (age and sex), clinical presentation (chest pain, dyspnea, palpitation, syncope, diarrhea, and upper respiratory tract infection symptoms), comorbidities (hypertension, diabetes mellitus, asthma, and smoking), laboratory variables (blood urea nitrogen [BUN], creatinine, estimated glomerular filtration rate, hemoglobin, white blood cell count [WBC], neutrophil count, lymphocyte count, C-reactive protein [CRP], albumin, peak hs-cTn, and peak CK-MB), electrocardiographic variables (heart rate, PR depression, ST elevation, ST depression, and T-wave inversion), echocardiographic variables (left ventricular ejection fraction [LVEF] and pericardial fluid), cardiac magnetic resonance variables (late gadolinium enhancement [LGE], LGE pattern, and myocardial edema), and medical therapies (beta blockers, renin–angiotensin system inhibitors [RASi], mineralocorticoid receptor antagonists [MRA], ibuprofen, colchicine, acetylsalicylic acid, and corticosteroids) were extracted from the electronic healthcare system.

Seasons were classified as winter, spring, summer, and autumn based on the calendar date of presentation. Winter was defined as the period from 1 December to 28 February; spring as 1 March to 31 May; summer as 1 June to 31 August; and autumn as 1 September to 30 November.

The admission electrocardiogram records were assessed based on several criteria: ST elevation occurring after the J point in two contiguous leads, with cut-off values of ≥0.2 mV for men and ≥0.15 mV for women in leads V2–V3, or ≥0.1 mV in all other leads. ST depression was defined as ≥0.1 mV measured at 80 ms from the J point. T-wave inversion was categorized as ≥0.1 mV deep in two or more leads, excluding lead aVR. T-wave inversion was determined by a negative amplitude of ≥1 mm in at least two contiguous leads [9].

Left ventricular ejection fraction was calculated using the Teichholz method on transthoracic echocardiography.

The late gadolinium enhancement pattern was defined according to the American Heart Association 17-segment model [10]. The LGE pattern was classified as multisegmental when present in ≥2 segments, at least involving the subendocardium in a distribution that did not conform to a single coronary territory.

The primary endpoint was recurrent myocarditis up to 2 years of follow-up.

The study protocol was reviewed and approved by Dokuz Eylül University Ethics Committee for Non-Interventional Research (protocol code 2024/41-02 and date of approval 11 December 2024). All procedures were conducted in strict adherence to the ethical principles outlined in the Declaration of Helsinki.

### 2.2. Statistical Analysis

All statistical analyses were performed using the institutional SPSS 31.0 version (SPSS Inc., Chicago, IL, USA). The distribution of continuous variables was assessed using the Kolmogorov–Smirnov test. Variables that were not normally distributed are presented as median (interquartile range) and were compared using the Mann–Whitney U test, whereas normally distributed variables are presented as mean ± standard deviation and compared using Student’s *t*-test. Categorical variables were evaluated using the chi-square test for the comparisons of baseline characteristics across patients stratified by the presence or absence of myocarditis recurrence. Comparisons of baseline characteristics across patients stratified by the presence or absence of myocarditis recurrence were carried out employing the Mann–Whitney U test for continuous variables, whereas categorical variables were evaluated using the chi-square test. To further investigate the significant overall association, post hoc tests were conducted using adjusted standardized residuals. Cells with an adjusted residual IzI ≥ 1.96 were considered significant at the 0.05 level. Continuous variables were reported as medians with quartiles (Q) 1 and Q3. Categorical variables were presented as counts and percentages. To delineate independent predictors of recurrent myocarditis, binary logistic regression analysis was employed. For this analysis, we created two models. Model 1 included age, sex, chest pain, dyspnea, pericardial fluid, CRP, peak hs-cTn, peak CK-MB, Hb, WBC, neutrophil count, lymphocyte count, PR depression, ST depression, ST elevation, T inversion, LVEF, LGE status, and pharmacological exposures, including colchicine usage, ibuprofen usage, and steroid usage. Model 2 incorporated covariates including age, sex, chest pain, dyspnea, pericardial fluid, CRP, peak hs-cTn, peak CK-MB, Hb, WBC, neutrophil count, lymphocyte count, PR depression, ST depression, ST elevation, T inversion, LVEF, LGE status, ibuprofen usage, steroid usage, and colchicine usage regarding usage time. We assessed the association of potentially significant factors, including ST depression, LGE, and optimal time of colchicine usage, between the patients with recurrent myocarditis versus those without recurrent myocarditis using multivariate Cox regression analysis, and hazard ratios (HRs) were reported with 95% confidence intervals (CIs). Cox regression analysis was adjusted for age, sex, chest pain, dyspnea, pericardial fluid, CRP, peak hs-cTn, peak CK-MB, Hb, WBC, neutrophil count, lymphocyte count, PR depression, ST depression, ST elevation, T inversion, LVEF, LGE status, ibuprofen usage, steroid usage, and colchicine usage regarding usage time.

## 3. Results

Among the 162 patients with myocarditis, the recurrence rate was 12.3%. The most common presentation was chest pain, followed by dyspnea. Although patients with recurrent myocarditis tend to present more frequently with shortness of breath, there was no statistically significant difference in the rates of the most common presentations between the two groups (for chest pain: 90.0% vs. 90.1%, *p* = 0.984; for dyspnea: 15.0% vs. 4.9%, *p* = 0.080). Patients who exhibited a myocarditis episode in the winter had a higher prevalence of recurrence than those in other seasons (IzI score: 3.4). In contrast, myocarditis in the spring was associated with a lower rate of recurrent myocarditis (IzI score: 2.4). Patients with recurrent myocarditis exhibited a lower neutrophil percentage (56.54 ± 15.06 vs. 63.43 ± 13.40, *p* = 0.036) at admission than patients without recurrent myocarditis. The presence of ST depression in the initial electrocardiogram was much more common in patients with recurrent myocarditis than in those without recurrent myocarditis (30.0% vs. 4.9%, *p* < 0.001). The median time from the index event to CMR was 7 days, with interquartile ranges of 3–14 days. LGE was present in 17 of 162 patients (10.49%). LGE was more common in patients with recurrent myocarditis (25.0% vs. 8.5%, *p* = 0.024), with a multisegmental LGE pattern involving the subendocardium being more frequent than in those without recurrent myocarditis (20.0% vs. 4.2%, IzI score = 2.7). Patients with recurrent myocarditis received a shorter duration of colchicine treatment than patients without recurrent myocarditis (2.50 months [1.00–2.50] vs. 3.00 months [2.50–3.50], *p* = 0.017) (Table 1).

According to Model 1, binary logistic regression analysis identified dyspnea (OR = 10.324, 95%CI = 1.040–102.459, *p* = 0.046), the presence of ST depression on the initial electrocardiogram (OR = 14.954, 95%CI = 2.529–88.432, *p* = 0.003), and LGE (OR = 7.231, 95%CI = 1.279–40.867, *p* = 0.025) as independent predictors of recurrence (Table 2). When colchicine use of at least 3 months was incorporated into the model, ST depression (OR = 14.469, 95%CI = 2.416–86.673, *p* = 0.003), LGE (OR = 10.362, 95%CI = 1.614–66.549, *p* = 0.014), and colchicine use of at least 3 months (OR = 0.295, 95%CI = 0.098–0.891, *p* = 0.030) emerged as independent predictors of recurrence (Table 3).

**Figure 1:**
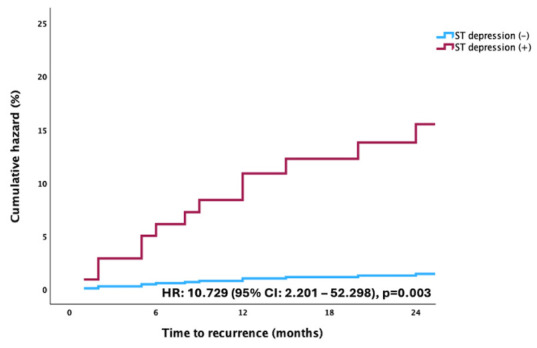
Cox regression curves for the cumulative hazard of myocarditis recurrence according to the presence of ST-segment depression. The abscissa (x-axis) represents the time to recurrence in months, and the ordinate (y-axis) represents the cumulative hazard expressed as a percentage (%). The blue line denotes patients without ST-segment depression (ST depression [−]), and the maroon line denotes patients with ST-segment depression (ST depression [+]); the (−) and (+) symbols indicate the absence and presence of the finding, respectively. Curves are step functions derived from the Cox proportional hazards model. The hazard ratio (HR), 95% confidence interval (95%CI), and *p*-value for the comparison between groups are displayed within the plot.

**Figure 2:**
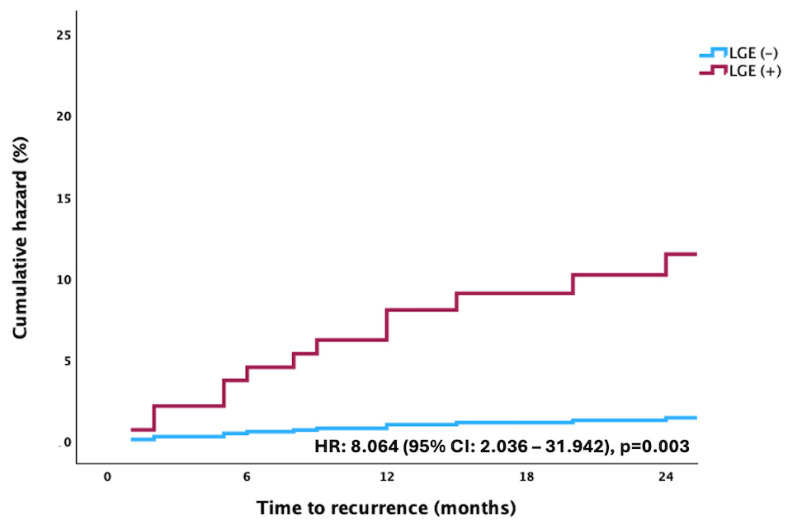
Cox regression curves for the cumulative hazard of myocarditis recurrence according to the presence of late gadolinium enhancement. The abscissa (x-axis) represents the time to recurrence in months, and the ordinate (y-axis) represents the cumulative hazard expressed as a percentage (%). The blue line denotes patients without LGE (LGE [−]), and the maroon line denotes patients with LGE (LGE [+]); the (−) and (+) symbols indicate the absence and presence of LGE, respectively. Curves are step functions derived from the Cox proportional hazards model. The hazard ratio (HR), 95% confidence interval (95%CI), and *p*-value for the comparison between groups are displayed within the plot. LGE, late gadolinium enhancement.

**Figure 3:**
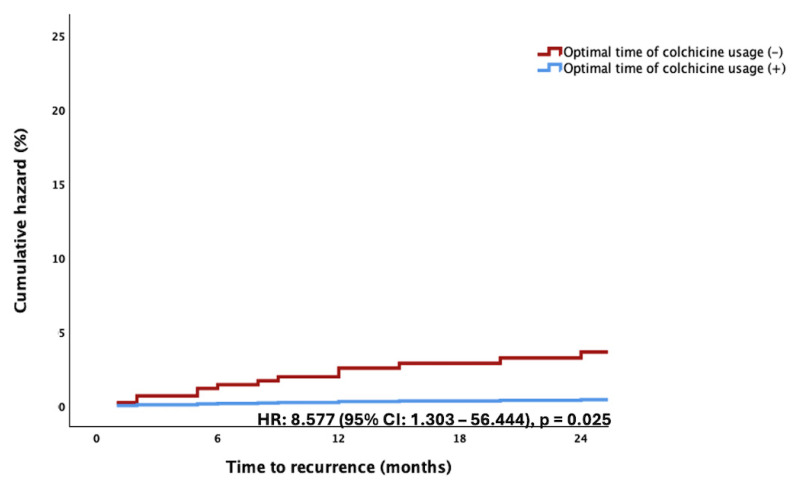
Cox regression curves for the cumulative hazard of myocarditis recurrence according to the time of colchicine usage. The abscissa (x-axis) represents the time to recurrence in months, and the ordinate (y-axis) represents the cumulative hazard expressed as a percentage (%). The maroon line denotes patients who did not receive colchicine within the optimal time window (optimal time of colchicine usage [−]), and the blue line denotes patients who received colchicine within the optimal time window (optimal time of colchicine usage [+]); the (−) and (+) symbols indicate, respectively, the absence and presence of colchicine initiation within the predefined optimal period. Curves are step functions derived from the Cox proportional hazards model. The hazard ratio (HR), 95% confidence interval (95%CI), and *p*-value for the comparison between groups are displayed within the plot.

The presence of ST depression on the admission electrocardiogram was associated with a higher risk of the development of recurrence relative to patients without ST depression (HR = 10.729, 95%CI = 2.201–52.298, *p* = 0.003) (Figure 1). In addition, the presence of LGE was associated with an increased risk of recurrence, relative to cases without LGE (HR = 8.064, 95%CI = 2.036–31.942, *p* = 0.003) (Figure 2). In contrast, colchicine use for at least 3 months was associated with a reduced risk of recurrence compared to cessation of colchicine (HR = 8.577, 95%CI = 1.303–56.444, *p* = 0.025) (Figure 3).

## 4. Discussion

In the present study, myocarditis recurrence was observed in 12.3% of patients with myocarditis during a 2-year follow-up, with a median time to recurrence of 13.5 months, consistent with previously reported recurrence rates of 10–15% in registry-based cohorts [5,11,12]. The Finnish registry reported an overall recurrence rate of 10.3%. However, the cumulative recurrence rate was 5.5% during the first 30 days and 7.3% over 7.2 months, with longer follow-up time correlating with higher recurrence rates [5]. Our study had a relatively prolonged follow-up time, which may indicate the importance of extended follow-up protocols beyond the acute phase, a point of particular clinical relevance given current guideline recommendations that largely focus on short-term management [13]. Notably, the recurrence window extending beyond 1-year follow-up indicates that patients who appear clinically stable during early follow-up visits may still possess residual myocardial vulnerability, potentially driven by persistent low-grade immune activation rather than overt inflammatory relapse.

Research directly examining seasonal patterns in myocarditis is limited but includes large registry and global burden analyses. Overall findings suggest only weak or no clear seasonality in incidence, but temperature, especially cold, is a relevant risk factor for myocarditis burden and outcomes [14]. To date, available literature has focused on the seasonality of index myocarditis episodes and related inflammatory conditions, leaving the temporal patterns of recurrent myocarditis largely unrecognized [14,15]. Our study indicates that the risk of recurrence is significantly higher among patients whose initial episode occurred in winter, whereas a spring onset was associated with a lower recurrence rate. This variation may be attributable to seasonal differences in viral etiology; winter-associated pathogens may exhibit greater pathogenicity, potentially leading to more sustained activation of proinflammatory pathways and subsequent myocardial vulnerability.

The clinical trajectory of myocarditis is often unpredictable; however, our findings identify ST-segment depression on the admission electrocardiogram as the most robust independent predictor of recurrent myocarditis. This prognostic significance remained consistent across both multivariate logistic regression models and time-to-event analyses, marking a critical departure from the classically recognized ST-elevation pattern. While an ST elevation pattern typically reflects a localized subepicardial region, ST depression is less frequently observed but may signify a more deleterious pathophysiological state, leading to extensive myocardial involvement and depressed ventricular function [9,16,17]. Such extensive myocardial involvement not only facilitates ventricular dysfunction—as evidenced by the higher prevalence of ST depression in patients with LVEF < 50% after a myocarditis attack reported by Younis et al.—but also serves as an early sentinel sign of fulminant progression [9,18,19]. We hypothesize that the ST depression pattern represents a diffuse transmural, mid-myocardial or subendocardial injury, likely driven by an increased immune-mediated response to viral insult. Crucially, the presence of ST depression on admission may identify a high-risk phenotype characterized by a greater systemic inflammatory burden and an underlying immunological predisposition to chronic recurrent inflammation. Recognizing this electrocardiographic marker is therefore vital for the early identification of patients at risk for long-term recurrence, allowing for more intensive monitoring and tailored therapeutic intervention.

In our cohort, LGE status was associated with subsequent recurrence, with affected patients carrying a markedly higher cumulative hazard than those without LGE. This association is biologically coherent. LGE has been explained by the increase in interstitial space due to interstitial edema or tissue infiltration of inflammatory cells in patients with acute myocarditis [20,21]. In general, it develops in the subepicardial region with variable extension to the rest of the myocardial thickness, and the identification of LGE on CMR signifies myocardial necrosis and/or fibrosis [21]. That is likely why previous studies have shown the presence and persistence of LGE as a sign of poor outcomes in myocarditis [22,23,24]. A study by Filippetti also showed that the presence of LGE after acute myocarditis was associated with mortality, recurrence, and hospitalization due to cardiovascular causes [25]. Beyond its role as a marker of fibrosis, its presence may reflect an incomplete resolution of the acute inflammatory process, with potential implications specifically for recurrence risk. The pattern of LGE has been proposed as a surrogate for the degree of residual myocardial injury following the index episode, and emerging evidence suggests that both the presence and the quantitative burden of LGE correlate with the likelihood of subsequent inflammatory reactivation, as in our study [26]. Fibrotic or necrotic myocardial tissue may serve as a nidus for perpetuating local immune activation by sustaining antigen presentation and promoting the recruitment of autoreactive T-lymphocytes, thereby establishing a self-reinforcing cycle of myocardial injury that lowers the threshold for recurrent episodes [27]. Furthermore, structural remodeling associated with LGE-defined fibrosis may alter the local tissue microenvironment in a manner that predisposes to recurrent episodes of inflammation or heart failure, ultimately impacting the overall cardiac function and patient prognosis [28]. All these observations may imply that LGE presence and pattern may represent not merely a scar but also a possible indicator of ongoing myocardial vulnerability to recurrence of myocarditis. In addition, LGE was present in 10.49% of our patients, a proportion below that of many acute myocarditis series [29]. This likely relates to the predominance of milder and uncomplicated cases in our cohort. Additionally, the majority of patients in our cohort, accounting for 93.2%, regularly received colchicine at the time of CMR. Such treatment may attenuate myocardial inflammation, potentially influencing LGE findings.

Colchicine is a lipophilic tricyclic alkaloid derived from Colchicum autumnale with a long-established anti-inflammatory role. Its benefit in pericardial and myopericardial disease is thought to stem from its ability to disrupt microtubule assembly and to concentrate within leukocytes—particularly granulocytes—where intracellular concentrations can exceed those in plasma more than 16-fold, even at the low oral doses used in clinical practice [30]. Through this mechanism, it impairs neutrophil activation and migration and interferes with assembly of the NLRP3 inflammasome, attenuating the downstream release of IL-1β and IL-18. These properties underlie its efficacy across a range of inflammatory conditions, including gout, familial Mediterranean fever, recurrent pericarditis, and Behçet disease and, more recently, in the reduction of cardiovascular events in atherosclerotic disease [31,32].

Clinical evidence supporting colchicine use in pure myocarditis is still evolving; however, contemporary data indicate potential benefit in myopericarditis, especially in reducing recurrences when the pericardium is involved. Notably, colchicine was associated with a ~60% lower recurrence rate in a large cohort of patients with first-episode pericarditis and myocardial involvement [33]. This is supported by real-world multicenter data showing improved three-month hard endpoints in patients with acute myocarditis treated within 14 days of presentation [34]. From a pathophysiological perspective, the activation of the NLRP3 inflammasome in the myocardium plays a central role in the pathogenesis of acute myocarditis, triggering a rapid inflammatory cascade mediated by IL-1β and IL-18. Targeting this signaling axis offers a compelling therapeutic strategy for the acute phase of the disease. Potential candidates include colchicine and specific anti-IL-1 agents; however, translating these theoretical benefits into clinical practice is currently constrained by a paucity of large-scale human evidence [13]. Crucially, an insufficient duration of colchicine therapy may facilitate the reactivation of the NLRP3 inflammasome pathway, potentially predisposing patients to clinical recurrence. In addition, colchicine is metabolized by CYP3A4 and transported by p-glycoprotein and is cleared mainly through hepatic and renal routes. Coadministration with enzyme inducers such as rifampin or carbamazepine can lower colchicine exposure and may thereby attenuate its therapeutic efficiency [35].

### Limitations

The present study has several limitations that warrant consideration. First, its retrospective and single-center design may introduce inherent selection bias and limit the generalizability of our findings to broader, more diverse patient populations. Second, the relatively small sample size resulted in wide confidence intervals for certain variables; therefore, our results should be interpreted as hypothesis-generating and require validation in larger, multicenter prospective cohorts. In particular, the multivariable models included a relatively large number of covariates for only 20 recurrence events, which confers a high risk of overfitting and likely explains these wide confidence intervals; the effect-size estimates should therefore be regarded as exploratory. Third, the findings reflect young adult patients; hence, children or elderly patients might have different courses. At the time of CMR, 151 (93.2%) patients were receiving colchicine, and we could not formally account for the effect of ongoing anti-inflammatory therapy on tissue characterization. LVEF was assessed using the Teichholz method, which derives volumes from linear left ventricular dimensions and assumes a symmetrical ellipsoid geometry; this approach may be less accurate than volumetric methods. Finally, as no deaths occurred during follow-up and only a small number of patients experienced recurrence, a survival analysis of arrhythmic events, recurrent myocarditis, and cardiac death was not statistically feasible.

## 5. Conclusions

The presence of ST-segment depression on the initial electrocardiogram and LGE pattern on CMR at a median of 7 days (IQR: 3–14) after the index event may serve as potent, independent predictors of recurrence in patients with acute myocarditis. Our findings suggest that these acute-phase markers are associated with a significantly higher risk of relapse, highlighting their utility in early risk stratification. Furthermore, maintaining colchicine therapy for a duration of at least three months was associated with a lower likelihood of recurrent episodes. These results suggest that identifying high-risk clinical and imaging phenotypes at admission may allow for a more personalized therapeutic approach, where ensuring an optimal duration of anti-inflammatory treatment may help stabilize the myocardial inflammatory milieu and improve long-term clinical outcomes. Finally, given the retrospective design and modest sample size, these findings should be interpreted with caution and confirmed in larger prospective studies with more robust designs.

## Figures and Tables

**Table 1:** The comparison of baseline characteristics in patients with myocarditis in terms of recurrence.

	Recurrence (+) (n = 20)	Recurrence (−) (n = 142)	*p*
Age (years)	27.00 (21.00–33.75)	24.50 (20.00–30.00)	0.400
Sex (female/male)	5/15	18/124	0.139
Season			0.004
Summer (n, %, IzI)	3 (15.0) (0.9)	34 (23.9) (0.9)	
Spring (n, %, IzI)	1 (5.0) (2.4)	44 (31.0) (2.4)	
Winter (n, %, IzI)	11 (55.0) (3.4)	29 (20.4) (3.4)	
Autumn (n, %, IzI)	5 (25.0) (0.0)	35 (24.6) (0.0)	
Clinical presentation			
Chest pain (n, %)	18 (90.0)	128 (90.1)	0.984
Dyspnea (n, %)	3 (15.0)	7 (4.9)	0.080
Palpitation (n, %)	2 (10.0)	9 (6.3)	0.542
Syncope (n, %)	0 (0.0)	3 (2.1)	0.512
Diarrhea (n, %)	4 (20.0)	28 (19.7)	0.976
Upper respiratory tract infection symptoms (n,%)	10 (50.0)	78 (54.9)	0.679
Comorbidities			
Hypertension (n, %)	1 (5.0)	3 (2.1)	0.436
Diabetes mellitus (n, %)	0 (0.0)	1 (0.7)	0.707
Asthma (n, %)	0 (0.0)	2 (1.4)	0.593
Smoking (n, %)	7 (35.0)	32 (22.5)	0.222
Laboratory variables			
BUN (mg/dL)	11.00 (8.35–13.00)	12.00 (10.10–14.15)	0.099
Cr (mg/dL)	0.77 (0.61–0.90)	0.80 (0.70–0.90)	0.337
eGFR (mL/min/1.73 m^2^)	116.50 (100.75–126.00)	120.00 (106.50–120.00)	0.687
Hemoglobin (g/dL)	13.95 (12.62–15.00)	14.40 (13.25–15.25)	0.292
WBC (µL)	8.40 (6.50–10.50)	8.80 (7.05–11.30)	0.449
Neutrophil count (%)	56.54 ± 15.06	63.43 ± 13.40	0.036
Lymphocyte count (%)	27.74 ± 8.84	23.50 ± 9.62	0.064
C-Reactive Protein (mg/dL)	43.50 (12.60–80.95)	44.55 (13.60–101.30)	0.982
Albumin (g/dL)	4.08 (3.80–4.38)	4.00 (3.70–4.26)	0.403
Peak hs-cTn (ng/mL)	15,072.50 (2449.25–35,761.75)	29,164.50 (6702.50–92,174.25)	0.098
Peak CK-MB (U/L)	24.75 (5.15–82.20)	23.35 (8.75–56.37)	0.761
Electrocardiographic variables			
Heart rate (bpm)	78.50 (72.75–87.50)	78.00 (70.75–88.00)	0.980
PR depression (n, %)	4 (20.0)	21 (14.8)	0.546
ST elevation (n, %)	6 (30.0)	49 (34.5)	0.690
ST depression (n, %)	6 (30.0)	7 (4.9)	<0.001
T inversion (n, %)	3 (15.0)	18 (12.7)	0.772
Echocardiographic variables			
LVEF (%)	60.00 (60.00–60.00)	60.00 (60.00–60.00)	0.350
Pericardial fluid (n, %)	1 (5.0)	8 (5.6)	0.908
Cardiac MR variables			
Myocardial edema (n, %)	2 (10.0)	5 (3.5)	0.182
Presence of LGE (n, %)	5 (25.0)	12 (8.5)	0.024
LGE pattern			0.017
None (n, %, IzI)	16 (80.0) (1.6)	130 (91.5) (1.6)	
Localized subepicardial (n, %, IzI)	1 (5.0) (0.9)	6 (4.2) (0.9)	
Multisegmental LGE (n, %, IzI)	4 (20.0) (2.7)	6 (4.2) (2.7)	
Medications			
Beta blockers (n, %)	13 (65.0)	101 (71.1)	0.574
RASi (n, %)	12 (60.0)	85 (59.9)	0.990
MRA (n, %)	0 (0.0)	1 (0.7)	0.707
Colchicine (n, %)	15 (75.0)	95 (66.9)	0.468
Ibuprofen (n, %)	17 (85.0)	94 (66.2)	0.090
Acetylsalicylic acid (n, %)	3 (15.0)	18 (12.7)	0.772
Corticosteroid (n, %)	0 (0.0)	1 (0.7)	0.707
Duration of colchicine usage (months)	2.50 (1.00–2.50)	3.00 (2.50–3.50)	0.017

BUN, blood urea nitrogen; Cr, creatinine; eGFR, estimated glomerular filtration rate; WBC, white blood cell; hs-cTn, high-sensitivity cardiac troponin; CK-MB, creatinine kinase-MB; LVEF, left ventricular ejection fraction; LGE, late gadolinium enhancement; MR, magnetic resonance; RASi, renin–angiotensin system inhibitors; MRA, mineralocorticoid receptor antagonists.

**Table 2:** Independent predictors of the development of recurrence in myocarditis in Model 1*.

	OR	95%CI	*p*
Dyspnea	10.324	1.040–102.459	0.046
ST depression	14.954	2.529–88.432	0.003
LGE	7.231	1.279–40.867	0.025

OR, odds ratio; CI, confidence interval; LGE, late gadolinium enhancement. * Model 1 included age, sex, chest pain, dyspnea, pericardial fluid, CRP, peak hs-cTn, peak CK-MB, Hb, WBC, neutrophil count, lymphocyte count, PR depression, ST depression, ST elevation, T inversion, LVEF, LGE status, and pharmacological exposures, including colchicine usage, ibuprofen usage, and steroid usage.

**Table 3:** Independent predictors of recurrence in myocarditis in Model 2*.

	OR	95%CI	*p*
ST depression	14.469	2.416–86.673	0.003
LGE	10.362	1.614–66.549	0.014
Colchicine usage at least 3 months	0.295	0.098–0.891	0.030

OR, odds ratio; CI, confidence interval; LGE, late gadolinium enhancement. * Model 2 included age, sex, chest pain, dyspnea, pericardial fluid, CRP, peak hs-cTn, peak CK-MB, Hb, WBC, neutrophil count, lymphocyte count, PR depression, ST depression, ST elevation, T inversion, LVEF, LGE status, ibuprofen usage, steroid usage and colchicine usage regarding usage time.

## Data Availability

The data presented in this study are available on request from the corresponding author.

## Abbreviations

The following abbreviations are used in this manuscript:

ICD-10	International Classification of Diseases, 10th Revision
CMR	Cardiac Magnetic Resonance
OR	Odds Ratio
CI	Confidence Interval
LGE	Late Gadolinium Enhancement
HR	Hazard Ratio
CK-MB	Creatine Kinase-MB
BUN	Blood Urea Nitrogen
WBC	White Blood Cell
CRP	C-Reactive Protein
LVEF	Left Ventricular Ejection Fraction
RASi	Renin–Angiotensin System Inhibitors
MRA	Mineralocorticoid Receptor Antagonists
MR	Magnetic Resonance
SPSS	Statistical Package for the Social Sciences
Hb	Hemoglobin
bpm	Beats Per Minute
mV	Millivolt
ms	Millisecond
aVR	Augmented Vector Right
hs-cTn	High-Sensitivity Cardiac Troponin
ESC	European Society of Cardiology
AEPC	Association for European Paediatric and Congenital Cardiology
EACTS	European Association for Cardio-Thoracic Surgery
NLRP3	NOD-like receptor family pyrin domain-containing 3
IL-1β	Interleukin-1 beta
IL-18	Interleukin-18
JACC	Journal of the American College of Cardiology
MRI	Magnetic Resonance Imaging
eGFR	Estimated Glomerular Filtration Rate
ng/mL	Nanogram per Milliliter
mg/dL	Milligram per Deciliter
U/L	Units per Liter
